# Responsible application of artificial intelligence in health care

**DOI:** 10.17159/sajs.2023/14889

**Published:** 2023-05-30

**Authors:** Adetayo E. Obasa, Andrea C. Palk

**Affiliations:** 1 Centre for Medical Ethics and Law, WHO Bioethics Collaborating Centre, Department of Medicine, Stellenbosch University, Cape Town, South Africa; 2 Unit for Bioethics, Centre for Applied Ethics, Philosophy Department, Stellenbosch University, Stellenbosch, South Africa

**Keywords:** artificial intelligence, responsible application, algorithm biases, ethical governance and regulation

## Introduction

Artificial intelligence (AI) can broadly be defined as the computational simulation of complex intellectual processes associated with intelligent human behaviour, such as learning, decision-making, problem-solving, executing tasks and self-correction.^1–3^ While the application of AI has widespread potential, its possibilities in health care are particularly significant, with research findings indicating that these technologies can already outperform humans in key healthcare tasks. For example, AI-powered machines are assisting radiologists in timeously identifying malignant tumours.^4^ The introduction of AI in the healthcare sector is primarily aimed at supporting the move towards precision medicine, including ensuring more efficient and accurate diagnoses and treatment plans. This will also have the benefit of relieving clinicians from the burden of mundane tasks. In this regard, AI technologies were successfully used during the COVID-19 pandemic to assist decision-making about prioritisation and allocation of scarce resources.^5^ While the introduction of AI in the healthcare sector is primarily aimed at improving service delivery within the industry^6^, the impact it will have on the healthcare sector as a whole, and on patient well-being in particular, will depend on how AI is developed, applied and regulated. Related to these are several ethical concerns that require urgent and continued attention.

First, to perform a given task with precision and efficiency, AI systems require access to extensive data sets. Within the healthcare context, these data sets are patient health information that would have been obtained from private and public hospitals, including government entities. This raises privacy concerns relating to data security as well as to ensuring that the appropriate consent to use data has been sought. Second, given human involvement in the initial training and learning of these systems, there are concerns that existing human prejudices and biases may inadvertently be introduced, leading to algorithmic, and consequently, decision-making biases. This has implications for health equity. Third, AI systems might make errors as part of the process of learning and becoming more efficient. If such systems improve to the extent that they can operate autonomously, we may have to reconfigure our models of responsibility and liability to accommodate such errors. These concerns regarding AI in health care are by no means exhaustive, but we regard them as particularly salient. Moreover, they imply the need for responsible and effective governance and regulation informed by a multidisciplinary and collaborative approach that considers the full array of ethical, legal, social, and economic implications of the use of AI technologies.^7^ In this Perspective, we discuss each of these concerns and provide some suggestions for ensuring responsible AI in health care.

### Data security, privacy and appropriate consent

Ethical AI includes respecting privacy as a fundamental value and right which in turn requires data security and protection.^8^ In South Africa, the *Protection of Personal Information Act* (POPIA) balances the right to protection of privacy, access to information and freedom of expression.^9^ This is pertinent given that for AI to function optimally in the healthcare sector, it requires access to extensive personal biometric information and data. However, POPIA does not accommodate all the specificities and challenges posed by the use of AI in health care. With the new reality of big data, mass quantities of patient data and personal data would be required by big tech companies to train and build algorithms. Although the data would be de-identified, the risk of reidentification remains plausible. Recent studies have shown how computational strategies can be used to reidentify individuals in health data repositories managed by both public and private institutions.^10,11^ One such study found that an algorithm could be used to reidentify 85.6% of adults and 69.8% of children in a physical activity cohort study “despite data aggregation and removal of protected health information”^12^. Insofar as the possibility of reidentification poses a significant obstacle to privacy, there is a need for new and improved data regulations that bolster this value and right. With the rapid pace of technology development, there are gaps in regulation and oversight that should be addressed through an innovative and multidisciplinary approach.

A related concern is how to ensure that appropriate models of consent have been used to obtain permission for the use of personal patient data, given that AI systems require access to vast data sets. The challenge here is ensuring that individual patients understand how their data might be used and the risk of reidentification, both requirements for meaningful consent. Moreover, as AI systems develop further, and are able to perform increasingly complex procedures, securing consent may prove challenging. While a sufficiently informative explanation of AI-enabled procedures would be necessary to ensure meaningful consent, the possibility of mistrust or fear of such technologies would require consideration. This implies that more studies are needed, in contexts in which such systems might be used, in order to ascertain optimum ways of communicating information and risks regarding these complex technologies.

### Algorithm biases and health equity

As mentioned above, access to vast data sets is crucial for the optimum functionality of AI, and for the process of machine learning and algorithm development, in particular. Therefore, if the data set itself is biased, this bias is transferred to the model that learns from the data. There is evidence that algorithm bias has already found its way into some AI devices; for example, pulse oximeters which have lower accuracy for populations with non-European ancestry due to the associated algorithms drawing on data sets comprised predominantly from populations of European ancestry.^13^ This raises distinct concerns about equity in health care. Biases fall into three main categories. First, bias could occur when skewed or misrepresentative data are fed as training data into an algorithm, for instance, data sets that exclude or underrepresent vulnerable populations, as is the case in the above example. Second, bias could occur due to malfunction or faulty algorithms. Third, bias could be introduced due to human prejudice informed by erroneous assumptions. In Africa, limited high-quality electronic data due to non-uniform or incomplete data sets could undermine data-oriented technologies and further exacerbate bias. Concerns about algorithmic inclusivity and the perpetuation of such biases are particularly urgent given that populations with African ancestry, across the globe, and in Africa, in particular, continue to be negatively impacted and harmed by ongoing prejudice. In clinical contexts in which AI is involved in diagnoses or providing predictions about the best possible treatment outcomes, biases in algorithmic processes could lead to serious harms related to misdiagnoses or inappropriate treatment. The responsible use of AI requires that its deployment in health care must be free from bias, and data ethics governance should be established to oversee software and algorithm development.^14^

### ‘Black box’ AI systems, trust and responsibility

Machine learning refers to the system of coded algorithms by which engineers inform artificial intelligence systems what to learn, what rules to apply to the learning process and the fundamental principles to apply. However, in the case of certain kinds of machine learning, these rules are not always fixed, they can be changed by the machine itself.^15^ Machine learning is commonly used in precision medicine to predict what treatment protocols will succeed based on various patient attributes and the treatment context.^16^ More complex forms of machine learning involve deep learning or neural network models with several layers of features and variables that predict outcomes. For example, a typical application of deep learning in health care is the recognition of potentially cancerous lesions in radiology images.

In clinical contexts there are concerns about the more complex forms of machine learning techniques, particularly the so-called ‘black box’ systems. The concern here is that black box systems are characterised by “opacity, complexity, and unpredictability” with the result that it is not possible to ascertain the process by which these systems deliver their output.^17^ While such systems are highly efficient, the possibility of errors is also a precondition of part of the learning process, in the same way that human beings learn more effectively through the allowance of error.^15^ Black box systems raise numerous ethical concerns, including explicability and accuracy, patient–clinician trust and broader questions regarding responsibility and liability in the case of errors or decisions that produce harmful consequences. In terms of the former, trade-offs might be required between increasing accuracy (at the cost of explainability) and enhancing a system’s explainability (which may reduce its accuracy).^18^ However, the degree of necessary explicability depends on the context and the risk involved. When there is a high risk of harm or negative outcomes associated with the decisions of such systems, we should be able to ascertain a full understanding of the decision-making process of the system. This implies that black box systems, for which such an explanation is not possible, should not be used with procedures that carry such high risk.

Currently, AI technologies support clinicians in decision-making, rather than operating autonomously; however, insofar as these systems improve and are able to operate independently, the transfer of decision-making from human agents to AI will elicit considerable ethical and legal concerns. Given that the law is configured in terms of the rights and obligations of human persons, an argument can be made that these rights should not be solely subjected to automated devices, especially when their decisions could have dire consequences.^19,20^ In South Africa, the da Vinci Xi fourth-generation system, one of the most advanced surgical robots in the world, is currently used by surgeons to perform robotic-assisted minimally invasive surgery in two public hospitals and several private hosiptals.^21^ This system has been built drawing on knowledge gained over the past two decades, ensuring substantial improvements in design and performance; its precision and accuracy cannot be overemphasised. While da Vinci is not fully autonomous, there is a possibility that future iterations might be deemed capable of independently performing specific tasks, carrying out decision-making processes, and proposing and validating strategies. Various ethical challenges will need to be addressed by regulatory bodies before this possibility is realised. As mentioned above, these include informed consent related challenges but also possibly a need to reconfigure our frameworks of responsibility to account for such autonomous systems as well as our legal frameworks in terms of liability for errors that might be made during procedures or associated harms.

Moreover, to foster trust and transparency, these systems might require the capacity to be sensitive to both ethical and social values in various multicultural contexts, and to justify their output, not only in the case of errors but in general. This would of course depend on the nature and purpose of the system. Trust is fundamental to the clinician–patient relationship insofar as the success of most medical interventions depends on it. As evidenced by previous abuses of trust in clinical and research contexts, this relationship is tenuous. While doctor–patient trust could be conferred to AI systems, any small failure in AI could significantly erode public confidence in health care. Once again, these challenges indicate a need for a regulatory framework that protects the safety of end users and ensures that the development of these devices is informed by a concern for fundamental human principles and values.

### Ethical governance and regulation

The report on *Ethics & Governance of Artificial Intelligence for Health* published by the World Health Organization in 2021 offers an excellent and practical resource for responsible development, design, use and regulation of AI.^22^ The guiding principles suggested in the report emphasise that the use, governance and regulation of AI should promote autonomy, well-being, trust, accountability, and equity, whilst being sustainable.^22^

In the context of considering ethical AI in health care, the notion of responsibility is fundamental. This includes both retrospective responsibility and prospective responsibility. The former is relevant in the case of dealing with errors that might be made by such systems, implying accountability or the need to be able to understand and explain the decisions of such systems, including any errors. In cases where harm is caused by an AI system in healthcare contexts, we should ensure that human beings are meaningfully involved in a way that we can identify parties who can be held accountable and responsible. However, the implication here is that completely autonomous AI systems that employ black box processes should not be used in certain healthcare contexts, given that such systems are not appropriate targets of our ascriptions of responsibility and accountability. Prospective responsibility requires that all stakeholders assume the duty to ensure the ethical roll out of AI. Responsible AI also underscores the significant role that educational interventions can play to ensure widespread knowledge and awareness and promote public acceptability and participation. Developers and manufactures of these devices must also be accountable to regulatory bodies and the public. Furthermore, there is a need for a regulatory framework mechanism to ensure that algorithm processes involved in AI systems meet declared ethical standards and expectations, such as the World Health Organization’s guidelines.^22^

## Conclusion

Given the enormous potential of AI to improve health care and enhance health outcomes in other areas, there will undoubtedly be an increase in the use of such systems over the next few decades. Addressing the above concerns will require ongoing ethical discussion, good governance and robust regulation. As argued by Jonas^23^, the development and application of science and technology should be grounded in recognition of the responsibility we bear to future generations. In the case of AI, we must govern and regulate it with awareness of the impact of our decisions on the well-being not only of all human beings who currently live, but also of those in the future.

## Significance:

The responsible application of artificial intelligence (AI) in health care is crucial as it has the potential to revolutionise medical practices. AI technologies can analyse medical data, identify patterns, and generate insights that can inform clinical decision-making, improve patient outcomes, and reduce healthcare costs. However, the ethical, legal, and social implications of AI in health care must be considered to ensure that its implementation is safe, transparent, and equitable. It is essential to prioritise the responsible application of these technologies to maximise their benefits and minimise potential harm. As AI continues to advance, its responsible application will play a vital role in shaping the future of health care.

## References

[1] Jha S, Topol EJ. Adapting to artificial intelligence. JAMA. 2016;316:2353–2354. 10.1001/jama.2016.17438 27898975

[2] Topol EJ. High-performance medicine: The convergence of human and artificial intelligence. Nat Med. 2019;25:44–56. 10.1038/s41591-018-0300-7 30617339

[3] Korteling JE (Hans), Van de Boer-Visschedijk GC, Blankendaal RAM, Boonekamp RC, Eikelboom AR. Human- versus artificial intelligence. Front Artif Intell. 2021;4. 10.3389/frai.2021.622364 PMC8108480 33981990

[4] McBee MP, Awan OA, Colucci AT, Ghobadi CW, Kadom N, Kansagra AP, Deep learning in radiology. Acad Radiol. 2018;25:1472–1480. 10.1016/j.acra.2018.02.018 29606338

[5] Singh JA, Moodley K. Critical care triaging in the shadow of COVID-19: Ethics considerations. S Afr Med J. 2020;110:355–359. 10.7196/SAMJ.2020v110i6.14842 32657716

[6] Yu KH, Beam AL, Kohane IS. Artificial intelligence in healthcare. Nat Biomed Eng. 2018;2:719–731. 10.1038/s41551-018-0305-z 31015651

[7] Dubber MD, Pasquale F, Das S, editors. The Oxford handbook of ethics of AI. New York: Oxford University Press; 2020. 10.1093/oxfordhb/9780190067397.001.0001

[8] Jobin A, Ienca M, Vayena E. The global landscape of AI ethics guidelines. Nat Mach Intell. 2019;1:389–399. 10.1038/s42256-019-0088-2

[9] Protection of Personal Information Act (POPI Act). Available from: https://popia.co.za/.

[10] Check Hayden E. Privacy loophole found in genetic databases. Nature. 2013. 10.1038/nature.2013.12237

[11] Gymrek M, McGuire AL, Golan D, Halperin E, Erlich Y. Identifying personal genomes by surname inference. Science. 2013;339:321–324. 10.1126/science.1229566 23329047

[12] Na L, Yang C, Lo CC, Zhao F, Fukuoka Y, Aswani A. Feasibility of reidentifying individuals in large national physical activity data sets from which protected health information has been removed with use of machine learning. JAMA Netw Open. 2018;1, e186040. 10.1001/jamanetworkopen.2018.6040 PMC6324329 30646312

[13] Tobin MJ, Jubran A. Pulse oximetry, racial bias and statistical bias. Ann Intensive Care. 2022;12:2. 10.1186/s13613-021-00974-7 34981267 PMC8723900

[14] Vyas DA, Eisenstein LG, Jones DS. Hidden in plain sight – reconsidering the use of race correction in clinical algorithms. N Engl J Med. 2020;383:874–882. 10.1056/NEJMms2004740 32853499

[15] Matthias A. The responsibility gap: Ascribing responsibility for the actions of learning automata. Ethics Inf Technol. 2004;6:175–183. 10.1007/s10676-004-3422-1

[16] Lee SI, Celik S, Logsdon BA, Lundberg SM, Martins TJ, Oehler VG, A machine learning approach to integrate big data for precision medicine in acute myeloid leukemia. Nat Commun. 2018;9:42. 10.1038/s41467-017-02465-5 29298978 PMC5752671

[17] Santoni de Sio F, Mecacci G. Four responsibility gaps with artificial intelligence: Why they matter and how to address them. Philos Technol. 2021;34:1057–1084. 10.1007/s13347-021-00450-x

[18] London AJ. Artificial intelligence and black-box medical decisions: Accuracy versus explainability. Hastings Cent Rep. 2019;49:15–21. 10.1002/hast.973 30790315

[19] Shickel B, Loftus TJ, Adhikari L, Ozrazgat-Baslanti T, Bihorac A, Rashidi P. DeepSOFA: A continuous acuity score for critically ill patients using clinically interpretable deep learning. Sci Rep. 2019;9:1879. 10.1038/s41598-019-38491-0 30755689 PMC6372608

[20] Vincent JL, Moreno R, Takala J, Willatts S, De Mendonça A, Bruining H, The SOFA (Sepsis-related Organ Failure Assessment) score to describe organ dysfunction/failure. Intensive Care Med. 1996;22:707–710. 10.1007/BF01709751 8844239

[21] Solomons L. Medical renaissance: Tygerberg becomes first public hospital to use da Vinci robot for surgery. News 24. 23 February 2022. Available from: https://www.news24.com/news24/southafrica/news/medical-renaissance-tygerberg-becomes-first-public-hospital-to-use-da-vinci-robot-for-surgery-20220223

[22] World Health Organization (WHO). Health workforce. Geneva: WHO; 2021 [cited 2022 Sep 12]. Available from: https://www.who.int/health-topics/health-workforce#tab=tab_1

[23] Jonas H. The imperative of responsibility: In search of an ethics for technological age. Chicago, IL: University of Chicago Press; 1984.

